# Molecular Biomarkers in Prediction of High-Grade Transformation and Outcome in Patients with Follicular Lymphoma: A Comprehensive Systemic Review

**DOI:** 10.3390/ijms252011179

**Published:** 2024-10-17

**Authors:** Marie Hairing Enemark, Jonas Klejs Hemmingsen, Maja Lund Jensen, Robert Kridel, Maja Ludvigsen

**Affiliations:** 1 Department of Hematology, Aarhus University Hospital, 8200 Aarhus N, Denmark; mariem@rm.dk (M.H.E.); johemm@rm.dk (J.K.H.); majalj@rm.dk (M.L.J.); 2 Department of Clinical Medicine, Aarhus University, 8000 Aarhus C, Denmark; 3 Princess Margaret Cancer Center, University Health Network, Toronto, ON M5G 2C4, Canada; robert.kridel@uhn.ca

**Keywords:** follicular lymphoma, molecular biomarkers, histological transformation, disease outcome

## Abstract

Follicular lymphoma (FL) is the most prevalent indolent B-cell lymphoma entity, often characterized by the t(14;18) *BCL2-IGH* translocation. The malignancy represents a clinically and biologically highly heterogeneous disease. Most patients have favorable prognoses; however, despite therapeutic advancements, the disease remains incurable, with recurrent relapses or early disease progression. Moreover, transformation to an aggressive histology, most often diffuse large-B-cell lymphoma, remains a critical event in the disease course, which is associated with poor outcomes. Understanding the individual patient’s risk of transformation remains challenging, which has motivated much research on novel biomarkers within the past four decades. This review systematically assessed the research on molecular biomarkers in FL transformation and outcome. Following the PRISMA guidelines for systemic reviews, the PubMed database was searched for English articles published from January 1984 through September 2024, yielding 6769 results. The identified publications were carefully screened and reviewed, of which 283 original papers met the inclusion criteria. The included studies focused on investigating molecular biomarkers as predictors of transformation or as prognostic markers of time-related endpoints (survival, progression, etc.). The effects of each biomarker were categorized based on their impact on prognosis or risk of transformation as none, favorable, or inferior. The biomarkers included genetic abnormalities, gene expression, microRNAs, markers of B cells/FL tumor cells, markers of the tumor microenvironment, and soluble biomarkers. This comprehensive review provides an overview of the research conducted in the past four decades, underscoring the persistent challenge in risk anticipation of FL patients.

## 1. Introduction

Follicular lymphoma (FL) represents the most common indolent lymphoma subtype. The malignant cells arise from B cells, which have often acquired a t(14;18) *BCL2-IGH* translocation [1,2,3,4,5,6]. With the exception of truly localized low-grade disease, FL is regarded as an incurable condition with a natural history of recurrent relapses. Nevertheless, therapeutic advances in the past decades have improved the prognosis markedly, with reported median survival times approaching two decades [7]. In contrast to the generally favorable clinical behavior of the disease, a significant portion of patients experience early disease progression, rapid treatment refractoriness, and/or histological transformation to a more aggressive lymphoma histology, most often diffuse large-B-cell lymphoma (DLBCL) [3]. The transformation event is accompanied by aggressive clinical behavior and markedly poor outcomes, reflecting the therapeutic implications of the high-grade lymphoma [2,5,8,9,10]. The past four decades of research have led to an improved understanding of the underlying biology of FL and its transformation; however, to this date no clinicopathological nor molecular marker has been able to unequivocally mirror the risk of transformation in FL [6]. Thus, despite the improved therapeutic regimens currently available for FL, the essential challenge of identifying FL patients with high-risk disease at the individual patient level remains. In the attempt to identify high-risk FL patients upfront, the search for novel biomarkers has long been of interest in FL research [3,5,11]. The long natural history and typically indolent behavior of FL provide opportunities to combine clinical and biological data to determine patient prognosis. Any biomarker harboring information on the risk of subsequent transformation already at the time of initial FL diagnosis would have the potential to aid treatment strategy and reduce mortality and morbidity [5].

Over the past four decades, several retrospective studies have assessed prognostic biomarkers for outcomes and transformation in FL [5]. The subject of this review was to systematically assess the research on molecular biomarkers in the transformation of FL. As transformation remains the leading cause of FL-related mortality, biomarker studies of time-related endpoints (overall survival, event-free survival, etc.) were also included in the study, ultimately aiming at a comprehensive overview of molecular biomarkers investigated in transformation and outcomes in FL.

## 2. Literature Search

**Figure 1:**
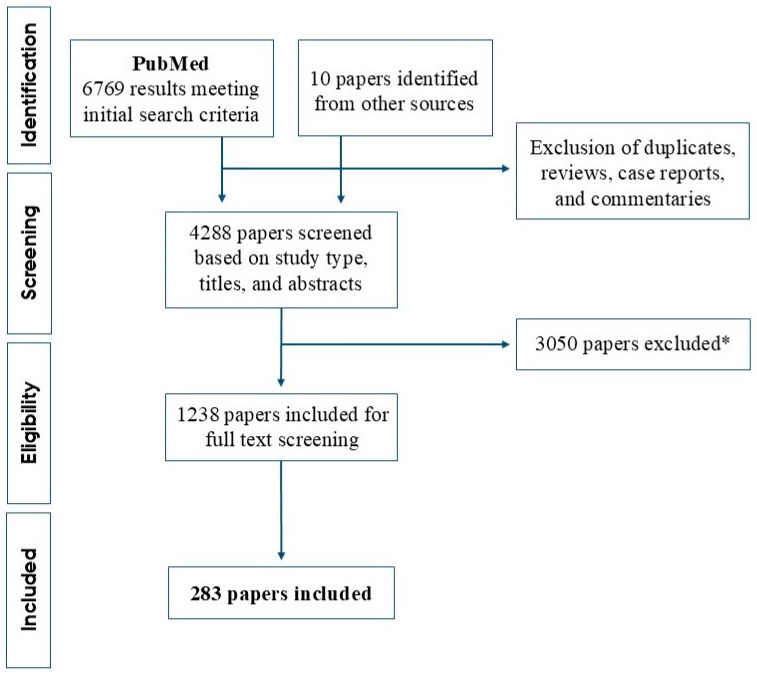
**PRISMA flow diagram.** The search strategy for the present review. * Review papers, case reports, studies of a non-FL study population, studies of relapse/refractory FL, studies of non-human tissues (i.e., animal models, cell line studies), studies with no molecular biomarkers evaluated, and otherwise irrelevant papers were excluded.

The review was conducted according to the PRISMA 2020 guidelines [12,13]. The PubMed database was searched to identify publications relevant to the topic. All review processes were performed using Covidence (Covidence systematic review software, Veritas Health Innovation, Melbourne, Australia. Available at www.covidence.org (assessed on 7 October 2024)), which is a web-based platform used to facilitate, e.g., screening and data extraction. Initial search criteria were restricted to studies written in English and published from January 1984 through September 2024. PubMed was searched with the inclusion of medical subject headings (MESH) terms using the search string (follicular lymphoma OR follicular lymphoma[MESH]) AND (outcome OR prognosis OR survival OR progression OR transformation), yielding 6769 results (Figure 1). The search option to exclude reviews, case reports, and commentaries was then applied. This resulted in a total of 4288 publications after the removal of duplicates. The identified publications were carefully screened by three reviewers (MBE, JKH, and MLJ) based on the type of study, titles, and abstracts, which left 1238 studies for full-text review. Included studies had to be original research studying molecular markers in pre-therapeutic samples in relation to transformation and/or prognosis of FL. Thus, we excluded the following: (i) review papers, (ii) case reports, (iii) studies of a non-follicular lymphoma study population, (iv) studies of relapsed/refractory FL, (v) studies of non-human tissues (i.e., animal models, cell line studies), (vi) studies with no molecular biomarkers evaluated, and (vii) otherwise irrelevant papers (e.g., sample sizes insufficient for statistical analysis). For more homogenous extraction, papers investigating pediatric FL and cutaneous FL were excluded. Moreover, commonly described markers such as β2-microglobulin, hemoglobin, lactate dehydrogenase (LDH), albumin, C reactive protein, serum immunoglobulins (Igs), Ki67, and the t(14;18)/*BCL2* rearrangements were not included. We did not discriminate in regard to the analytical method used in the studies. Reference lists of included studies and relevant review papers were searched to identify any studies that may have been missed by the database search, which resulted in the inclusion of an additional 10 studies. Full-text screening and data extraction were performed by three reviewers (MBE, JKH, and MLJ), after which a total of 283 studies were eligible for inclusion in the final review. A PRISMA flow chart illustrating the inclusion process is presented in Figure 1.

The included studies focused on molecular biomarkers, investigating their potential role as predictors of transformation or as prognostic markers of time-related endpoints. The effect of each biomarker on prognosis or risk of transformation, respectively, were divided into three classes, i.e.,

(i) None, if the biomarker was investigated but no statistically significant impact on prognosis or risk of transformation was reported,
(ii) Favorable, if the biomarker was associated with superior prognosis or lower risk of transformation,
(iii) Inferior, if the biomarker was associated with worse prognosis or higher risk of transformation.

Favorable or inferior associations between biomarker and outcome were committed to reports of significance levels of *p* < 0.05. Trending results not reaching statistical significance at this setting were included in the class “none”.

## 3. Review of Studies of Putative Biomarkers

Studies investigating molecular biomarkers in relation to the risk of subsequent transformation, disease progression, and/or survival were reviewed. According to our search criteria, 283 papers were included for review (Figure 1). An increasing number of papers investigating molecular biomarkers in correlation with clinical outcomes were seen over time, with the majority of the included articles published in the last two decades. This was as expected, as molecular and scientific advancements, technologies, and possibilities have improved at a rapid pace during the study period, January 1983–September 2024.

For detailed study information, the analyzed markers have been separated into groups: (i) genetic abnormalities, Table 1, Table 2, Table 3 and Table 4; (ii) gene expression, Table 5, Table 6 and Table 7; (iii) microRNAs, Table 8; (iv) B cells/FL tumor cells, Table 9; (v) the tumor microenvironment, Table 10, Table 11, Table 12, Table 13, Table 14, Table 15 and Table 16; and (vi) soluble biomarkers, Table 17 and Table 18. As such, several tables have been subdivided to clarify their content. This separation into different biomarker groups was an attempt at simplifying the large amount of data, and thus, we advise the reader to keep in mind that several included biomarkers (e.g., different proteins) may be expressed in several cell types or function in other pathways than where they are listed in the tables. Moreover, we did not discriminate studies based on whether analyses were performed specifically on tumor cells or using bulk material, which may add to the diversity of the tables.

### 3.1. Genetic Abnormalities

The pathogenesis of FL is a complex, multistep process, which remains incompletely elucidated [4,6,14]. Accordingly, much research has focused on genetic abnormalities in FL. These abnormalities can involve changes in tumor suppressor genes, oncogenes, or genomic instability. Identifying such genetic abnormalities not only enhances our comprehension of complex diseases but also provides promising drug targets. Through whole-genome sequencing, targeted deep sequencing, single-nucleotide polymorphism arrays, and many more, chromosomal structural variants and mutational changes have been identified (Table 1, Table 2, Table 3 and Table 4).

**Table 1:** General genomic changes.

	Reported Risk of Transformation			Reported Prognostic Value		
	Favorable	Inferior	None	Favorable	Inferior	None
Genomic or karyotypic changes		[15]			[16,17,18,19,20]	[21,22,23]
Increasing number of mutations		[24,25]	[21]		[21,26,27,28,29,30]	[21,31,32,33,34]
M7-FLIPI [35]		[24]			[26,34,35,36]	[27,32,33,37,38,39,40]
*TNFRSF14-KMT2D-HIST1H1E*-FLIPI [41]		[41]				
DLBCL-like mutational status [42]		[42]				
TT genetic subtype (NFκB members and *TP53*) [43]					[43]	

#### 3.1.1. Cytogenetic Abnormalities

Cytogenetic abnormalities have provided valuable insights into the molecular mechanisms underlying FL pathogenesis and comprise significant implications for prognosis and treatment strategies. The most characteristic example in FL is the translocation t(14;18)(q32;q21), resulting in the constitutive expression of the anti-apoptotic BCL2 protein, which is present in around 85% of diagnosed cases and is considered a hallmark event of FL development [1,2,5,44]. Certain regional chromosomal imbalances and genomic or karyotypic changes have been linked to poor outcomes and an increased risk of transformation. However, it is not known whether their association with inferior outcomes could merely reflect a collectively increased genomic complexity, as the number of chromosomal alterations in itself has been reported to be correlated with adverse outcomes [5,15,16,17,18,19,20].

Further characterization of cytogenetic abnormalities has revealed specific chromosomal alterations associated with distinct clinical outcomes in FL. Chromosome 1 aberrations, such as the loss of 1p (encoding genes such as *NOTCH2*, *CD58*), and especially 1p36 (encoding genes such as *TNFRSF14*, *MTOR*, *ARID1A*, *C1QA*, *TP73*), are linked to inferior overall survival and an increased risk of transformation [18,33,45,46,47,48]. Losses on 3q27 (encoding genes such as *BCL6*) have also been reported linked to inferior outcomes [19]. Similarly, alterations in chromosome 6 are associated with varied prognostic implications. Loss of 6q (encoding genes such as *SESTRIN1*, *TNFAIP3*, *BLIMP1*) is generally associated with inferior OS and an increased risk of transformation, although there is conflicting evidence suggesting a favorable risk of transformation in one study [16,17,18,29,46,48,49,50]. Conversely, the gain of 6p (encoding genes such as *IRF4*, *PIM1*, *CCND3*, *CDKN1A*, *HIST1H1E*, *HLA-A/B/DR*) exhibits contradictory effects, with some studies indicating a favorable risk of transformation while others suggest an inferior prognosis. Gains on chromosomes 7 (*CARD11*, *EZH2*, *KMT2C*), 8q (*MYC*), 12q (*KMT2D*, *MDM2*, *STAT6*, *ATP6AP1*, *BCL7A*, *BTG1*), and 18 (*BCL2*), as well as losses on 16p (*CREBBP*, *SOCS1*) have been described with inferior outcomes regarding both transformation and time-related endpoints [16,20,22,33,46,49,51,52]. Furthermore, chromosome 17 abnormalities seem to play a crucial role in FL prognosis. Loss of 17p (encoding genes such as TP53) is consistently associated with inferior OS and an increased risk of transformation, while loss of 17q (encoding genes such as *CD79B*, *GNA13*, *STAT3*) also has been reported to contribute to inferior OS [18,19,20,46,47,48,53]. Conversely, gain of 17q has also been linked to an inferior risk of transformation and worse OS outcomes [20,46,54]. Loss of chromosome 22q (*EP300*) and gains of 21 (*BACH1*, *RUNX1*, *SCL19A1*) and X (*BTK*, *UBE2A*, *CXCR3*, *FOXP3*) have also been described, implicating several genes often reported in FL pathogenesis [16,19,20,47,49]. Interestingly, the above-mentioned genetic areas may share important common features. For instance, *TP73* and *TP53* are located on 1p36 and 17p, respectively [55,56]. *TP53* may be the most well-described tumor suppressor, while *TP73* is a member of the p53 tumor suppressor gene family. Loss of genetic material in these areas might result in the loss of key tumor suppressor functions, contributing to cancer development and progression. While *TP53* mutations themselves occur only in approximately 5–7% of FL, this could indicate other deregulation leading to the loss of tumor suppressor function aside from the commonly known *TP53* mutations and/or deletions [5,11,57].

Structural alterations involving *BCL6* have been associated with outcomes in FL, with *BCL6* translocations reported to confer an increased risk of transformation; however, studies examining the general prognosis of FL patients with *BCL6* rearrangements yield conflicting results [58,59,60,61]. Similarly, copy number changes affecting *BCL6* have been implicated in inferior OS and an increased risk of transformation, underscoring the need for further research to elucidate their precise role in FL pathogenesis and prognosis [62]. Indeed, the germinal center (GC)-specific BCL6 protein is critical in the FL biology, as it modulates the cell cycle, response to DNA damage, and anti-apoptotic molecules, which allows somatic hypermutation and class-switch recombination to proceed, altogether affecting gene expression that favors perpetuation of the GC B-cell state [63].

The comprehensive understanding of cytogenetic abnormalities in FL provides not just potential valuable prognostic markers but also cancer driver genes. However, to fully understand the potential, research efforts are warranted to unravel the complex genetic landscape of FL and identify novel therapeutic targets.

**Table 2:** Chromosomal abnormalities.

	Reported Risk of Transformation			Reported Prognostic Value			Reported Risk of Transformation			Reported Prognostic Value		
Chr	Losses on						Gains on					
	Favorable	Inferior	None	Favorable	Inferior	None	Favorable	Inferior	None	Favorable	Inferior	None
1p					[18,47,48]	[19]						[19]
1p36		[46]	[24]		[33,45,46]	[53]						
1q						[18,19]	[49]		[46]		[46,47]	[17,19]
2								[49]				[47]
2p						[18,47]			[64]		[20,49]	[64]
2q					[20]							
3p												[47]
3q						[47]		[49]			[49]	
3q27					[19]	[18]						
4						[47]						
4p					[53]							
4q					[18]	[17]						
5								[49]				[47,65]
5p									[46]		[46,49,50]	
5q						[18,53]					[49]	
6												
6p							[49]	[46]			[33]	[46,47]
6q	[49]	[18,46]	[24]		[16,17,18,46,48,50,65]	[19,47,53]						
7									[64]			[18,19,47,64,65]
7p									[46]		[22,46]	[17,51]
7q									[46]			[17,46]
8												[47]
8p						[51]						
8q		[66]			[20,66]			[46]	[64]		[16,46]	[64]
9p					[16,50]							
9q						[53]						
10p						[47]						
10q			[46]			[18,19,46,47,53]						[19]
11q											[50]	[51]
12											[22,47]	[18,19,65]
12p						[47]						[17]
12q								[46,49]	[64]		[49]	[18,46,64]
13					[65]							
13p						[47]						
13q						[17,18,19,53]						
15						[47,65]						
16p					[20]							
17p		[18]	[46]		[18,20,46,47,48,53]							[17]
17q					[19,47,53]	[18]		[46]			[20,46]	[47]
18											[52]	[18,19,47]
18p									[46]			[17,46]
18q						[47,53]			[46,64]		[33,51]	[17,46,64]
19p			[46]			[46]						
21								[49]			[19]	[47]
22						[47]						
22q			[46]		[20]	[46]						
X											[16,47,49]	[18,19,65]
Xp											[16]	[17]
Xq												[17]

#### 3.1.2. Gene Variants

The majority of studies investigating mutations in FL have focused on the general prognostic impact, meaning that specific information on gene mutation status in relation to transformation is more limited. In several studies, more than one mutational spot was analyzed. For specific variants in the listed genes, we refer the reader to the individual studies.

Some studies have reported an increasing overall mutational burden associated with the risk of transformation, while another study found no difference [21,24,25]. Similarly, several studies have found inferior prognosis in patients with an increased mutational burden, which not all studies have been able to replicate [21,26,27,28,29,31,32,33,34]. In general, transformed FL differs from preceding indolent FL by higher numbers of genetic aberrations, presenting with mutations of known putative oncogenes and tumor suppressor genes, which are not necessarily mutated at the time of diagnosis [5,67,68].

*BCL2 gene variants*. Apart from the hallmark t(14;18) involving the *BCL2* gene, *BCL2* variants have been reported to be correlated with transformation and outcome in four studies but not associated with outcome in others, which might be due to heterogenous methodologies, sequencing depth, considerations of non-coding/coding sequences, and more [21,26,33,38,51,52,69,70,71,72,73].

*BCL6 gene variants*. While *BCL6* translocations confer risk of transformation, it remains less described whether *BCL6* variants by themselves act as a marker of FL with increased propensity to transform. Indeed, several genes involved in constitutive BCL6 protein activation have been associated with lymphomagenesis (*BCL6*, *MEF2B*, *IRF4*, *IRF8*). *BCL6* variant itself has been reported with inferior risk of transformation, although several studies report no predictive value [21,73,74,75]. Interestingly, *IRF8* mutations have been reported with a favorable value regarding risk of transformation but with an inferior value on outcome [24,69]. The majority of studies on all four genes, however, have reported no prognostic value [21,26,27,33,38,52,73,74,75]. These inconclusive and differing observations could underpin differences in methodologies or specific mutation sites; either way, further investigation on the role of these genes might be worth exploring.

*Epigenetic modifiers*. Mutations in epigenetic modifiers are often described as key pathogenic factors of FL. These include mutations in histone modifiers (*KMT2D* (also known as *MLL2*), *CREBBP*, *EZH2*, *EP300*), members of linker histones (*HIST1H1B/C/D/E,* etc.), as well as SWI/SNF chromatin remodeling complex genes (*ARID1A*, *BCL7A*). In addition to being recurrently described in FL development, studies with a focus on *CREBBP*, *EP300*, and *BCL7A* variants have been reported yielding inferior outcomes [24,25,27,32,35,38,76]. Oppositely to the other investigated epigenetic genes, *EZH2* mutations have been described associated with favorable outcomes [26,35,52,77,78]. Contradictory to this, the effects on transformation or outcome have also been reported without predictive or prognostic value, as the association seems to be treatment-dependent [21,27,33,38,79,80]. Interestingly, reports of *ARID1A* variants have revealed favorable, inferior, and no impact on prognostics [21,26,27,33,35,51,69,81]. Mostly, investigations of different linker histones have reported no prognostic or predictive value, with the exception of a prognostic association with *HIST1H2AC* and *HIST1H1D* variants [21,26,33,38,69]. Furthermore, *HIST1H1E* variants have been reported conferring inferior risk of transformation [24,41]. As such, variants of epigenetic modifiers seem to be implicated in the pathogenesis of FL, but it still awaits further research into specific understanding of the mechanisms and, in particular, the predictive value.

**Table 3:** Other structural abnormalities.

	Reported Risk of Transformation			Reported Prognostic Value		
	Favorable	Inferior	None	Favorable	Inferior	None
**Uniparental disomy**						
Number of abnormalities					[64]	
1p36					[64]	
6p			[64]			[64]
6q			[64]			[64]
10q			[64]			[64]
12q			[64]			[64]
16p		[64]			[64]	
**Gene rearrangements**						
Number of structural rearrangements		[21]			[21]	
*BCL6*		[60]		[58]	[61]	[59]
*LAZ3*						[82]
*MYC*		[66,83]	[62]		[66]	[58,62,84,85]
**Copy number changes**						
*BCL2*			[62]			[62]
*BCL6*		[62]			[62]	
*IRF4*			[62]		[86]	
*MYC*			[62,66]		[62]	[66]

*Cell cycle*. Genes related to cell cycle progression, such as *TP53*, *MYC*, and *CDKN2A,* have also been widely investigated. Although not that frequently mutated in FL, *TP53* is one of the genes most strongly associated with transformation, and is frequently reported in tFL samples [6,32,34,57,87,88]. Similarly, *MYC* mutations are relatively rare at FL diagnosis, while they are often reported at tFL. The prognostic impact of specific *MYC* variants, however, remains a bit more unclear [21,73]. Some studies have correlated *MYC* rearrangements with both inferior risk of transformation and outcome, and case reports have also highlighted a rare subgroup of clinically relevant double-hit FLs with coexistence of *BCL2/MYC* rearrangements [66,83]. On the contrary, mutations in *CDKN2A* have been reported with inferior values on both transformation and outcome, making this an interesting target for further analysis [69,89,90].

*Immune-related and B-cell receptor signaling*. Much research has also been conducted focusing on immune-related and B-cell receptor signaling genes, including *FCGR2A/2B/3A*. Many different polymorphisms in these genes have been investigated, yielding results of favorable, inferior, and no prognostic impact, depending on the site of the mutation [28,91,92,93,94,95,96,97,98]. In the rituximab era, these genes are most definitely an interesting subject for investigation, as several of the rituximab anti-tumoral mechanisms rely on different FCγ receptors, e.g., antibody-dependent cell cytotoxicity and antibody-dependent phagocytosis [99,100,101,102].

Collectively, a myriad of single genetic variants has been analyzed, investigating predictive or prognostic value on transformation or outcome. Here in the text, a few have been described; however, many more interesting data are enclosed in Table 1, Table 2, Table 3 and Table 4.

*M7-FLIPI*. Pastore et al. described how weighted inclusion of the mutational status of seven genes commonly described in FL (*EZH2*, *ARID1A*, *MEF2B*, *EP300*, *FOXO1*, *CREBBP*, and *CARD11*) incorporated with the FLIPI score, named the M7-FLIPI score, improved risk stratification for failure-free survival. This score has later been investigated by several studies, with different reporting on predictive power. Roughly half of the studies were able to validate the inferior prognostic value of the score, including one study also reporting inferior impact on transformation, while the other half found no difference in outcome [24,26,27,32,33,34,35,36,37,38,39,40]. Differences have also been reported depending on the choice of treatment (e.g., bendamustine, obinutuzumab vs. CHOP/CVP). Noteworthy, one such study found that in a non-chemotherapy-containing regimen, the M7-FLIPI was not prognostic of outcome [39]. With the specific endpoint of progression of disease within 24 months (POD24), Jurinovic et al. analyzed the genes of the M7-FLIPI score, yielding a better stratification model, the POD24 prognostic index (POD24-PI) refined to include FLIPI as well as the mutational status of only three of the genes (*EP300*, *FOXO1*, and *EZH2*) [36].

**Table 4:** Specific genetic variants.

	Reported Risk of Transformation			Reported Prognostic Value				Reported Risk of Transformation			Reported Prognostic Value		
Gene	Favorable	Inferior	None	Favorable	Inferior	None	Gene	Favorable	Inferior	None	Favorable	Inferior	None
*ABL2*						[51]	*HSP27*						[73]
*ACTA*						[73]	*HSP40*						[73]
*ACTB*						[21,73]	*HTR2B*						[73]
*ADAM17*				[103]			*HVCN1*				[38]		[21]
*APEX1*						[104]	*ID2*						[73]
*ARHGEF1*						[21]	*IFNGR1*					[105]	[92]
*ARID1A*		[81]		[35]	[69]	[21,26,27,33,51]	*IGHV*		[106]				
*ARID1B*						[21,33]	*IGHV1*						[107]
*ATP6AP1*						[21]	*IGHV3*						[107]
*ATP6V1B2*						[21,33,38]	*IGHV4*						[107]
*B2M*		[41]			[21]	[27,32]	*IGHV5*					[107]	
*BACH2*					[21]		*IGHV6*						[107]
*BCL2*		[72]			[69,70,71,72]	[21,26,33,38,51,52,73]	*IGLL5*						[32,33]
*BCL6*		[75]				[21,73,74,75]	*IKZF3*					[21]	
*BCL7A*					[24]	[21,38]	*IL10*						[28,108]
*BCR*						[21]	*IL12A*					[108]	
*BHMT*						[104]	*IL12B*					[28,92]	
*BIM*					[109]		*IL13*					[28]	
*BMP6*						[73]	*IL16*						[28]
*BMP7*				[103]			*IL17A*						[108]
*BRCA1*						[104]	*IL17F*						[108]
*BRCA2*						[104]	*IL1RN*					[28]	[92]
*BTG1*					[21]	[27]	*IL2*				[92]	[28]	[108]
*BTG2*					[21]	[21,38]	*IL2RG*						[73]
*BTK*		[69]				[38]	*IL3*						[28]
*C1QA*				[110]	[110]		*IL4R*					[105]	[21,92,105]
*C1QB*						[111]	*IL5*					[28]	[105]
*C1QC*						[111]	*IL6*						[105]
*C1QTNF7*						[111]	*IL7R*						[105]
*C1RL*						[111]	*IL8*				[92]	[105]	[28,105]
*C1S*						[111]	*IL8RB*						[28]
*C2*						[111]	*IRF4*						[21]
*C3*				[111]			*IRF8*	[69]				[24]	[21,26,27,33,38]
*C3AR1*						[111]	*IL10*						[105]
*C4BPA*					[111]		*IL12B*						[105]
*C5*					[111]		*IL16*						[105]
*C5AR1*						[111]	*ITPKB*						[21]
*C6*						[111]	*JUN*						[73]
*C6orf15*		[112]			[112]	[92]	*KLHL6*						[21]
*C7*				[111]			*KMT2C*					[21]	
*C8B*						[111]	*KMT2D [MLL2]*		[41]	[25]			[21,26,33,38,51,52]
*C9*				[111]	[111]	[92]	*LGMN*				[48]		
*CARD11*					[35]	[21,26,27,33,38,52]	*LIG4*						[104]
*CARD15*						[105]	*LRRC7*						[21]
*CBS*						[104]	*LRRN3*						[38]
*CCDC129*						[38]	*MAP3K11*						[73]
*CCNB*						[73]	*MASP2*						[111]
*CCND3*					[71]	[21]	*MBD2*						[104]
*CCR2*					[105]		*MBL2*					[111]	
*CCR4*				[37]			*MDM2*			[113]			[113]
*CD46*					[92,111]		*MEF2B*						[21,27,38,52]
*CD55*				[111]	[92]		*MEF2C*				[21]		
*CD59*						[111]	*MGMT*						[104]
*CD69*				[48]			*MIF*					[105]	[92]
*CD79B*						[21,33,38]	*MINOR*						[73]
*CD83*						[21]	*MKI67*					[21]	
*CD8A*				[37,48]			*MLH1*					[97]	[104]
*CD8B*				[37]			*MSH2*						[104]
*CD93*						[111]	*MTHFD2*						[104]
*CDC2*						[73]	*MTHFR*				[92]	[104]	
*CDKN1A*						[73]	*MTHFS*						[104]
*CDKN2A*		[69]			[69,89,90]	[90]	*MTR*						[104]
*CFB*						[111]	*MTRR*						[104]
*CFD*						[111]	*MUC4*						[38]
*CFH*				[111]	[111]	[92]	*MYC*						[21,73]
*CFHR1*				[111]			*MYD88*					[21]	[32,52]
*CFHR5*					[111]	[92]	*MYOM2*						[21]
*CHI3L1*					[114]		*NBS1*						[104]
*CHD8*						[21]	*NCOR2*						[33]
*CIITA*						[21]	*NLRC5*						[21]
*CLU*						[111]	*NOTCH1*						[21]
*COL3A1*						[73]	*NOTCH2*		[24,41]				[21,33]
*CR1*					[111]		*NPM3*						[73]
*CR2*					[111]		*NR2F6*						[73]
*CREBBP*	[25,41]		[76]	[25]	[32,38,76]	[21,26,27,33,51,52]	*OVGL*						[73]
*CSMD3*		[41]											
*CTLA4*						[105]	*P2RY8*						[21]
*CTSS*				[115]		[21]	*PDCD4*						[73]
*CX3CR1*					[114]		*PIEZO1*						[73]
*CXCR3*				[37]			*PIM1*					[34]	[21,27,38]
*CXCR4*						[38]	*PLAU*						[73]
*CXCR5*		[116]	[116]		[116]	[116]	*POU2AF1*						[21,38]
*CYBA*						[97]	*POU2F2*						[38]
*CYHR1*						[33]	*PRF1*						[37]
*DEFB115*						[38]	*PRKCB*						[73]
*DNAH9*						[21]	*PRKCG*						[73]
*DTX1*		[24]				[21]	*PSMB1*				[91]		
*DUSP2*					[103]		*PSMB5*						[91]
*DUXA*						[38]	*PSMB8*						[91]
*E2FS*					[117]		*PSMB9*						[91]
*EBF1*						[21,27]	*RAD23B*						[104]
*EBF3*						[21]	*RAG1*						[104]
*EEF1A1*						[21]	*RFX5*						[21]
*EIF2B*						[73]	*RHOA*						[21]
*EML6*					[117]		*RHOH*					[21]	
*EOMES*				[37]			*RPS9*						[73]
*EP300*		[41]			[27,35]	[21,26,38,52]	*RRAGC*						[21]
*ERCC1*						[104]	*S1PR2*						[21]
*ERCC2*						[104]	*SELE*					[105]	[92]
*ERCC4*						[104]	*SERPING1*						[111]
*ERCC5*						[104]	*SGK1*						[21,27]
*EVI2A*						[21]	*SHMT1*						[104]
*EZH2*	[41]	[25]	[25,79]	[26,35,52,77,78]		[21,27,33,38,79,80]	*SLC19A1*						[104]
*FAS*					[21]	[27,52]	*LC25A23*						[33]
*FAT4*						[21]	*SMAD1*						[73]
*FCGR2A*				[28,91]		[91,92,93,94,95,96,97]	*SMARCA4*						[21,26]
*FCGR2B*				[95]	[95]	[95]	*SOCS1*					[21,32]	[27,33]
*FCGR3A*				[91,96,118]	[96]	[93,94,95,97,98]	*SORT1*						[33]
*FLT3LG*				[37]			*STAT3*						[21]
*FOXO1*					[26,34,35]	[27]	*STAT6*						[21,26,32,33,38,51]
*FPGS*						[104]	*TBX-21*				[37]		
*FTHFD*					[104]	[92]	*TCF3*						[21]
*GADD45B*				[103]			*TCN1*						[104]
*GALNT12*				[103]		[92]	*TGFB1*						[108]
*GAPDH*						[73]	*TGFBR1*						[108]
*GGH*					[104]	[92]	*TGFBR2*						[108]
*GNA13*		[69]				[21,26,27,38]	*TLE1*						[73]
*GNAI2*						[21]	*TNF/LTA*					[73]	[28]
*GSTA1*						[97]	*TNFAIP3*						[21,52]
*GSTM1*					[119]		*TNFRSF14*	[41]		[45]	[120]	[45]	[21,26,33,38,48,52]
*GSTT1*					[119]		*TP53*		[41,69]	[113]		[21,24,32,35,57,69,74,121]	[27,33,38,104,113]
*GZMM*				[37]			*TPTE2*						[38]
*GZMK*				[37]			*TYMS*						[104]
*HIST1H2AC*					[69]		*UBE2A*		[24,41]				
*HIST1H1B*						[21]	*UNC5C*						[21]
*HIST1H1C*						[21,33,38]	*USP44*					[117]	
*HIST1H1D*					[33]	[38]	*VEGFA*					[122]	
*HIST1H1E*		[24,41]				[21,26,33,38]	*VMA21*						[38]
*HIST1H2AM*						[21,38]	*WRN*						[104]
*HIST1H2BK*						[38]	*XBP1*					[21]	[27]
*HIST1H3G*						[38]	*XPB*					[73]	
*HIST2H2AC*						[38]	*XPC*						[104]
*HLA-A*					[123]		*XRCC1*						[104]
*HLA-B*				[123]			*XRCC2*						[104]
*HLA-C*						[123]	*XRCC3*						[104]
*HLA-DMB*						[21]	*XRCC4*						[104]
*HLA-DRA*						[73]	*YY-1*					[73]	
*HLA-DRB*				[123]			*ZFP36L1*						[21]
*HLA-DQA*						[73]	*ZFPC150*						[73]
*HLA-DQB1*			[112]			[112]	*ZFX*						[73]
*HNRNPU*						[38]	*ZNF608*						[38]
*HSF1*						[73]							

### 3.2. Gene Expression

*Outcome-associated gene expression profiles*. The malignant FL cells reside within a rich TME enveloped by nonmalignant immune bystander cells and critical immune components. The contribution of the TME to patient outcome was first described by Dave et al. [124], who demonstrated that immune-related gene expression profiles with a high expression of genes from FL tumor-associated macrophages was indicative of poor outcomes, which was later validated in an independent cohort [124]. However, other studies have later shown conflicting results, sometimes depending on incorporated treatment regimens, not able to reproduce the prognostic value (Table 5, Table 6 and Table 7) [53,125].

Another gene expression profiling study, performed by Huet et al. [126], defined a signature based on the expression of 23 genes characteristic of B-cell centroblasts that correlated with adverse outcomes in FL [126]. The adverse impact of this signature was later validated in independent cohorts [34,117,126,127].

*Transformation-associated gene expression profiles*. In continuation of B cell-related genes, Brodtkorb et al. [128] described a gene expression signature of NFκB-related genes, which were reported to be of prognostic value regarding both outcomes and subsequent transformation, which was later validated by Steen et al. [128,129].

Other gene expression signatures reporting unfavorably on the risk of transformation include an embryonic stem-cell-like signature [130]. Moreover, several signatures have reported favorable outcomes, including a GCB-like FL signature and two different T-cell signatures [37,125,131,132,133,134].

**Table 5:** Gene expression signatures.

	Reported Risk of Transformation			Reported Prognostic Value		
	Favorable	Inferior	None	Favorable	Inferior	None
Pluripotency/embryonic stem cell-like signature [130]		[130]		[130]		
23-GEP risk score [126]					[34,117,126,127]	[37,53]
ICA13 [126]					[126]	
33 gene-based ABC-like FL signature [131]					[131]	
33 gene-based GCB-like FL signature [131]				[131]		
T-cell associated immune infiltration signature [37]				[37]		
T effector signature [132]				[132]		[53]
T cell exhaustion signature [135]					[135]	
NFκB-linked signature [128]		[128,129]			[128,129]	
Somatic hypermutation signature [69]			[69]		[69]	
m6A score, low [136]					[136]	
FL loci risk score [133]				[133]		
MAP signature [32]					[32]	
STAT signature score [134]				[134]		
Immune-related 1 [124]				[124]		[53,125]
Immune-related 2 [124]					[124,137]	[53,125]
Localized-stage FL signature [125]				[125]		
TH17-axis related [138]					[138]	

*Expression of single genes*. Prognostic or predictive effects of the expression of single genes have also been performed, primarily investigating outcomes and less so transformation. Tobin et al. [139] performed a large, targeted gene expression analysis, where they showed differences in the expression of immune markers or immune checkpoints. Of particular note, low levels of immune markers would identify patients enriched for early progression. Here, the immune checkpoint molecule *PD-L2* was the marker with the highest accuracy [139]. Conversely, another study did not find this same correlation [53]. Single genes whose expression have been linked to subsequent transformation include B-cell/immune-related genes such as *BTK*, *CCL19*, *CCL20*, *CD101*, *CD138*, *CD2*, *CD69*, *CD9*, *CXCL1*, *CXCR6*, *GZM-K*, *IFN-γ*, *IGBP1*, *IL1R*, *LYN,* and more [128,140,141]. Many more studies have focused on outcomes and gene expression, with various reports of both favorable and inferior correlations.

At present, the assessment of these genes as well as the capacity to perform gene expression studies are not standardized at diagnosis, as some techniques are not readily available. Furthermore, while indeed interesting, on the way to understanding the underlying tumor biology, the expression signatures of dozens of genes may currently be more appropriate in research settings rather than in the clinical setting.

**Table 6:** Gene expression levels.

	Reported Risk of Transformation			Reported Prognostic Value				Reported Risk of Transformation			Reported Prognostic Value		
Gene	Favorable	Inferior	None	Favorable	Inferior	None	Gene	Favorable	Inferior	None	Favorable	Inferior	None
*ABHD6*				[137]			*KLRB1*		[140]				
*ACTB*						[142]	*KNT2C*						[143]
*ACTN1*						[142]	*KIAA0100*					[137]	
*AKAP12*						[142]	*KIAA0101*				[143]		
*AKIRIN1*				[137]			*KIAA0317*				[144]		
*AKT*					[145]		*KIAA1223*					[144]	
*ALDH1L1*						[137]	*KRT19*					[137]	
*ANP32E*				[143]			*LAG3*						[139]
*ARFGEF1*				[137]			*LEF1*						[142]
*ARPC2*				[137]			*LGMN*						[142]
*ASAP2*				[137]			*LIPA*		[140]				
*ATPAF2*				[137]			*LPP*				[137]		
*BCL-XL*					[146]	[145]	*LYN*	[140]					
*BCL6*						[145]	*MAL2*		[140]				
*BLCAP*				[144]			*MALAT1*			[141]		[141]	
*BLNK*				[144]			*MAP3K7*			[128]			
*BNIP2*					[137]		*MAPK1*						[142]
*BRI3BP*				[144]			*MARCO*					[147]	
*BTD*				[137]			*MED6*					[137]	
*BTK*		[128]					*MED8*					[137]	
*BTN2A3P*					[137]		*MLPH*					[144]	
*BUB1B*						[143]	*MMP9*					[148]	
*C1S*					[144]		*MOX2*					[144]	
*C1QR1*					[144]		*MYC*					[145]	
*C3AR1*						[142]	*MZB1*					[149]	
*C4B*					[144]		*NCF4*					[144]	
*CAV1*					[144]		*NEK2*						[142]
*CBFA2T2*					[137]		*NFIB*					[148]	
*CCL19*		[140]		[148]			*NFκB*						[145]
*CCL20*		[140]					*NGFRAP1*					[144]	
*CCL3*						[142]	*NK4*					[144]	
*CCL5*						[142]	*NPDC1*					[144]	
*CCL8*						[142]	*NSDHL*				[137]		
*CCNA2*				[143]			*PAICS*						[143]
*CCNB1*				[143]			*PBX1*		[140]				
*CCNB2*						[143]	*PD-1*						[139]
*CCND1*					[144]		*PD-L1*						[139]
*CCR1*					[142]		*PD-L2*				[139]		[53]
*CD101*		[140]					*PIM1*				[134]		
*CD11d*						[142]	*PLA2G2D*					[148]	
*CD137*						[139]	*PPP4R1*			[128]			
*CD138*		[140]					*PRB1*					[137]	
*CD19*						[142]	*PRH1*				[137]		
*CD2*		[140]				[142]	*PSMF1*				[137]		
*CD3*				[142,150]			*PTAFR*				[137]		
*CD31/* *PECAM1*					[151]		*PTEN*						[145]
*CD34*						[151]	*PTGDS*						[148]
*CD3D*						[142]	*PTP4A2*						[137]
*CD4*				[139]		[142]	*PTPRB*	[140]					
*CD47*						[142]	*PTPRC*	[140]					
*CD5*						[142]	*PTPRF*	[140]					
*CD6*						[142]	*PTPRM*					[144]	
*CD68*				[139]		[142]	*RAB27B*		[140]				
*CD69*		[140]				[142]	*RAB38*					(144)	
*CD7*						[139,142]	*RANBP9*					[137]	
*CD8*				[150]			*RET*					[144]	
*CD8A*						[139]	*RGL1*						[148]
*CD8B*						[142]	*RPS9*						[142]
*CD9*		[140]					*ROCK1*		[128]				
*CDC2*				[143]			*RRM2B*				[144]		
*CDC40*					[137]		*SEP-10*						[142]
*CDC42BPK*		[140]					*SH2D1A*		[140]				
*CDK2*						[142]	*SIRT5*				[137]		
*CDKN3*				[143]			*SLC21A9*					[144]	
*CKS1B*				[143]			*SLC24A2*					[137]	
*CRY1*					[144]		*SLC7A11*				[137]		
*CXCL1*		[140]					*SLP1*		[140]				
*CXCR6*		[140]					*SMAD1*					[147]	
*CXCL12*						[142]	*SNX9*					[144]	
*DAAM2*					[144]		*SOCS1*					[134]	
*DNAAF1*				[137]			*SOCS3*						[134]
*DUSP6*					[144]		*SPP1*					[144]	
*ELF3*		[140]					*SSI-3*					[144]	
*EPHA1*				[147]			*ST14*				[144]		
*EVA1B*					[137]		*STAT2*						[134]
*EZH2*						[77]	*STAT3*						[134]
*FASTKD1*			[128]				*STAT4*		[140]				[134,142]
*FCGR1A*						[142]	*STAT5a*				[134]		
*FOXP3*						[139]	*STAT6*						[134]
*FREB*				[144]			*TAB2*			[128]			
*FRYL*					[137]		*TAF12*				[137]		
*GAPDH*						[142]	*TBK1*			[128]			
*GEM*						[142]	*TCP10L*				[137]		
*GLE1L*				[144]			*TDRD12*				[137]		
*GMDS*						[143]	*TIA-1*		[140]				
*GZM-K*		[140]					*TIM3*						[139]
*H2BFB*				[144]			*TIMP3*						[148]
*H2BFG*				[144]			*TLR5*						[142]
*HMGB2*						[143]	*TM4SF1*		[140]				
*HMMR (RHAMM)*						[143]	*TMED7-TICAM2*		[128]				
*HSF2*				[137]			*TMEM70*					[137]	
*IDH3A*				[137]			*TMP3*		[140]				
*IDO1*				[132]			*TNF-alfa*				[139]		
*IF2B*						[142]	*TNFSF13B*					[144]	[142]
*IFITM1*					[144]		*TNFRSF14*					[152]	
*IFN-γ*	[140]						*TNFSF10*					[144]	
*IGBP1*		[128]					*TOP2A*						[143]
*IKBKG*			[128]				*TOP2B*				[137]		
*IL1R*		[140]					*TRBα*					[144]	
*IL2*						[134]	*TRIM37*		[128]				
*IL2Rα* *(CD25)*					[134]	[142]	*TRPM4*					[137]	
*IL4*				[134]			*TSC22D3*			[128]			
*IL4R*					[134]		*TSPAN7*				[137]		[142]
*IL7*					[134]		*TTLL3*				[137]		
*IL7R*						[134]	*TYROBP*					[144]	
*ILF3*						[142]	*UACA*					[144]	
*INPP5B*					[53]		*UBQLN1*				[144]		
*IRAK1*		[128]					*USP11*			[128]			
*ITK*				[137]		[142]	*VEGF*				[151]		
*JAK2*						[134]	*YAP1*					[148]	
*JUNB*					[144]		*ZNF230*				[137]		
*KLK10*					[137]								

**Table 7:** Epigenetic regulation.

	Reported Risk of Transformation			Reported Prognostic Value		
Methylation Status on	Favorable	Inferior	None	Favorable	Inferior	None
*DAPK*					[153,154]	
*MGMT*					[153]	
*p15*						[153]
*p16*						[153]

### 3.3. MicroRNAs

Despite their paucity, studies exploring microRNAs (miRNAs) have provided little evidence of their role in the prognosis and transformation of FL, and further research is needed to fully understand and exploit the potential of this field. miRNAs participate in the posttranscriptional regulation of gene expression by binding target mRNAs, resulting in their repression or degradation. The small RNA molecules have been emerging as biomarkers in cancer research and implicated in cancer pathogenesis, either as oncogenic modulators or tumor suppressors [155,156]. In recent years, miRNAs have become a recurrent theme in hematological malignancies, including leukemia, myeloma, and several B-cell lymphomas, including FL, DLBCL, and mantle cell lymphoma. However, they have not shown clear predictive potential [157,158,159]. Central to FL tumor biology and pathogenesis, several miRNAs are involved in the regulation of B cell biology pathways, including B cell development, B-cell receptor (BCR) and NFκB signaling, apoptotic regulation, as well as DNA damage response [159].

While studies investigating miRNA involvement in FL transformation are sparse, transformation seems to be, at least, partially related to miRNA regulation [6,156,160,161,162]. Musilova et al. reported five miRNAs enriched at transformation, including miR-150. From this, they proposed an MYC/miR-150/FOXP1 axis resulting in greater FOXP1 expression, a transcription factor involved in B cell development associated with inferior outcomes in both FL and DLBCL [6,162,163].

Future directions of research on miRNAs in FL requires a focus on elucidating the function and/or prognostic value of the transformation of FL and the impact of the small RNAs on the TME. Furthermore, the increasing evidence of the importance of miRNAs in lymphoma pathobiology have spurred an endeavor to develop novel miRNA-based therapeutics [159,160]. Despite developments within this field in recent years, we are far from understanding the gene networks regulated by miRNAs in B cells, and thus, further studies are warranted.

**Table 8:** miRNAs.

	Reported Risk of Transformation			Reported Prognostic Value		
	Favorable	Inferior	None	Favorable	Inferior	None
miR-217	[164]					
miR-221		[164]				
miR-222		[164]				
miR-223	[164]					
let-7i		[164]				
let-7b		[164]				
miR-150				[162]		
miR-7e-5p	[165]					

### 3.4. B Cells/FL Tumor Cells

The malignant cells of FL resemble germinal-center centroblasts and centrocytes that usually express pan-B-cell antigens such as CD19, CD20, CD22, and CD79a as well as CD10, BCL2, and BCL6 [1,2,7,166,167]. While BCL2 overexpression is essential for FL development, expression levels of the protein have been reported with different effects on outcome, though with the majority of studies reporting no prognostic impact [48,61,168,169,170,171,172,173,174,175,176,177]. Likewise, overexpression and constitutive activation of BCL6 is well described on FL; however, reports on any prognostic value remain inconsistent, with differences depending on intratumoral localization [60,61,169,171,172,177,178,179]. In addition to BCL2 overexpression, additional secondary genetic aberrations are commonly described in FL as a result of the genetic diversification of Ig genes by AID in the affinity maturation of the B-cell receptor. Regarding AID protein expression, although not restricted to B cells only, one study reported an inferior impact on the risk of transformation [72].

Notably, an inferior impact on the risk of transformation as well as outcome was reported by Carreras et al. on expression of the HVEM protein (encoded by the *TNFRSF14* gene, recurrently mutated in FL) [152]. The HVEM protein induces the activation or inactivation of B and T lymphocytes depending on its interaction with different ligands, one being the BTLA protein. When lost in the B cells, the HVEM–BTLA binding is disrupted, ultimately favoring mitogenic signaling in the B cell. Accordingly, in addition to inferior impact of HVEM expression, a favorable association with outcome was reported with increasing levels of BTLA [152].

Different aspects of the B-cell receptor have also been investigated, with only IgD expression yielding reports of prognostic value [61,172,180]. Furthermore, different essential B-cell transcription factors have been analyzed, including FOXP1 and PAX5, conferring reports of inferior impacts on outcome and transformation, respectively [162,163,181]. An inferior association with the risk of transformation and outcome was also reported on the IRF4 (also known as MUM1) protein expression, which is reflective of increased NFκB activation [60,182,183]. Given its role in enhancing BCR signaling, this could also potentially be a candidate for immunomodulatory drugs that downregulate IRF4.

**Table 9:** B cells/FL tumor cells.

	Reported Risk of Transformation			Reported Prognostic Value		
	Favorable	Inferior	None	Favorable	Inferior	None
**B cells**						
BCL2				[168]	[169]	[48,61,170,171,172,173,174,175,176,177]
pBCL2						[184]
CD19					[185]	
CD20				[169]		[142,186,187]
Interfollicular CD20			[140]			[58,188]
CD21						[177]
CD37					[189]	[189]
CD69					[190]	
CD79a						[169]
FOXP1					[162,163]	[171,179]
HVEM (TNFRSF14)		[152]			[152]	
Follicular HVEM					[152]	
Interfollicular HVEM					[152]	
MUM1 (IRF4)		[60]			[182,183]	[61,171,172,175]
OCT2						[169]
PAX5		[181]				[181]
**Germinal center cells**						
BCL6			[60]	[178]		[61,169,172,177]
Follicular BCL6						[171]
Interfollicular BCL6					[171]	[179]
CD10	[60]			[172,178]		[61,142,169,175,177,191]
Follicular CD10						[171,177]
Interfollicular CD10					[188]	[171,179]
CD10 negative				[192]		
CD75				[169]		
HGAL						[54]
Serpin A9/GCET1						[54]
**Immunoglobulins**						
IgA						[180]
IgD					[172]	
IgG						[180]
IgM						[180]
κ Ig light chain						[61]
λ Ig light chain						[61]
**Tumor phenotype**						
FL with features of pre-CSR, IgM^+^IgG^−^ memory B-cells		[193]				
FL with features of normal GC B-cells		[193]				
Phenotypic diversity among malignant B-cells		[193]				
CSR, class switch recombination; GC, germinal center.						

### 3.5. The Tumor Microenvironment

The malignant FL cells reside in an immunologically active tumor microenvironment (TME), and research has long been conducted in relation to the neoplastic versus nonmalignant bystander cells. Rather than inherent properties of the tumor cells themselves, infiltrates of macrophages, dendritic cells, T cell subsets, stromal cells, as well as a smaller proportion of neutrophils and natural killer (NK) cells have been proposed to influence FL growth and progression, ultimately determinative of the clinical behavior and prognosis [124,140,190,194]. The precise nature and role of these cell populations is still unclear, and published data on their predictive and prognostic significance remains highly contradictory, as seen in Table 10, Table 11, Table 12, Table 13, Table 14, Table 15 and Table 16. Specific cell subset populations have been correlated with a poor prognosis in some series, while reported with favorable or insignificant prognoses in others. Discrepancies in the studies might be explained by technical variations, which is often seen in the case of the interpretation of, e.g., immunohistochemical data, which many studies are based on. However, the differences may also reflect the underlying tumor heterogeneity of FL.

Included studies explored the relationship between immunological factors of the TME and risk of transformation or time-related survival endpoints.

Some studies investigated predictive and/or prognostic markers in relation to received treatment. In the case of some markers, even if they did not show predictive or prognostic value for the overall population, they may hold value for the treatment effects. This observation supports the explanation that variabilities in reported results may be explained by tumor heterogeneity and diversity in patients’ clinical characteristics and, ultimately, their treatment.

A plethora of different cellular markers, cellular subpopulations, and corresponding cellular mechanisms have been investigated in FL. Here, we highlight a number of examples; however, much more equally important research is shown in Table 10, Table 11, Table 12, Table 13, Table 14, Table 15 and Table 16.

#### 3.5.1. T Cells

Tumor-infiltrating T cells can be classified into several populations, including T_FH_s, T-regulatory cells (T_reg_s), and CD8^+^ T cells, which can further be divided into distinct subgroups based on functional distribution, including naïve, mature, effector, activation, exhausted, and memory cells.

*CD4^+^ and CD8^+^ T cell numbers*. A prominent feature of the FL TME is indeed the higher concentration of CD4^+^ T_FH_s and T_reg_s compared with CD8^+^ cytotoxic T cells. Further, this also holds value in prognostics of the disease course reflected by inferior outcomes correlated with a higher CD4/CD8 ratio [195,196], again reflecting their opposing roles in immune responses. Correspondingly, many studies have reported generally favorable outcomes correlated with cytotoxic T cell-related markers, including CD8 and granzyme B [52,117,185,187,196,197,198,199,200]. Interestingly, however, one study reported an inferior impact on transformation associated with increased CD8 expression [201].

CD4^+^ T cells in FL have been abundantly studied and are often characterized by localization within rather than between follicles, often reflective of T_FH_s. Especially, CD4 expression has been reported with high diversity regarding the impact on outcomes, even when divided into intratumoral compartments (i.e., whole-tumor, intrafollicular, interfollicular localization) [52,108,117,142,150,177,185,190,196,197,201,202,203,204,205,206,207]. Nonetheless, regarding the impact on transformation, intrafollicular expression of CD4 has been reported to be inferior, while interfollicular expression was associated with favorable values [140,201]. For both expression of CD8 and CD4, it is important to recognize that several distinct cellular subgroups exist, which may explain some of the discrepancies in the reports.

*CD4^+^FOXP3^+^ T_reg_s*. It is a central tenet of the clonal selection theory that lymphocyte repertoires are tolerized to self-antigens during their ontogeny [208]. Conventional CD4^+^CD25^+^FOXP3^+^ T_reg_s have been studied abundantly in FL, with many discrepancies in prognostic outcome, ranging from no effect through both favorable and inferior outcomes [52,108,117,150,152,168,171,175,183,190,196,197,201,203,205,206,209,210,211,212,213,214,215,216,217]. However, when investigated in relation to transformation, reports have been only of either inferior or no predictive value [140,196,201,205,209]. Furthermore, studies investigating the activation of T_reg_s (i.e., activated T*reg* phenotype, CD25 expression) have reported inferior or no effect on prognostic outcome [203,205,218,219,220]. Discrepancies in reported prognostic/predictive values may be related to the administered treatment. Indeed, de Jong et al. reported that the impact of FOXP3 and other microenvironmental factors was dependent on specific treatment protocols [190].

*Other regulatory T cells*. In recent years, other regulatory T cell subsets have been discovered. One such is CD8^+^FOXP3^+^ T regulatory cells. The CD8 compartment is analogous to a FOXP3^+^ population, which shares phenotypic aspects with the more common CD4^+^ T_reg_ counterpart [221]. A key feature of the CD8^+^FOXP3^+^ population is the relative scarcity compared with the more plentiful CD4^+^ population. The key reason for the paucity of research is the low frequency of CD8^+^FOXP3^+^ T cells. However, studies have shown the suppressive capacity of equivalent efficacy as CD4^+^FOXP3^+^ T_reg_s, especially potent in exerting class I-restricted repression, also potentially serving a unique function in the regulation of B-cell responses [221]. Another rather newly described population is the discovery of a subset of T follicular regulatory cells (T_FR_s), which share phenotypic characteristics with both conventional T_reg_s (CD4^+^CD25^+^FOXP3^+^) and T_FH_s (BCL6^+^ICOS+CXCR5^+^PD-1^+^) [222,223,224,225,226,227,228,229,230,231]. These constitute a discernable population of regulatory T cells that express the germinal center-defining transcription factor BCL6 and, thus, migrate to the follicles, where they exert a suppressive level of control on the maintenance of the GC immune homeostasis. The mere existence of such regulatory T cell subtypes is interesting in the context of FL pathology due to its known tumor-promoting TME. However, not much research has yet focused on these. Hagos et al. reported a favorable outcome with the CD8^+^FOXP3^+^ T_reg_ subset [216]. Investigation into T_FR_s remains most definitely warranted. Given the apparent reliability of FL on GC state perturbation, BCL6 expression, and TFH infiltration, knowledge of the cellular interplay between malignant B cells and different types of regulatory T cells would be of high interest.

**Table 10:** T cells.

	Reported Risk of Transformation			Reported Prognostic Value		
	Favorable	Inferior	None	Favorable	Inferior	None
CD3			[140,196,201]	[142,150,185,213,232,233,234]		[52,108,177,186,187,190,196,201,206,235,236]
Follicular CD3		[201,237]	[196]			[52,188,190,196,201,206]
Interfollicular CD3		[237]	[196]			[52,188,190,196,206]
CD5					[238,239]	
CD7			[196]	[142]		[177,196,203]
Follicular CD7			[196]			[196]
Interfollicular CD7			[196]			[196]
ZAP-70						[187]
CD4/CD8 ratio			[205]		[195,196]	[205]
CCR7				[232]		
GATA3				[195]		
**CD8^+^ T cells**						
CD8		[201]	[140,185,196]	[52,185,196,197,198]	[240]	[142,150,177,187,190,201,203,212]
Follicular CD8		[201]	[196]	[117,187]	[108]	[52,190,196,201,215]
Interfollicular CD8			[196]	[52,196]		[117,215]
CD8^+^CXCR5^+^				[241]		
Granzyme B			[196]	[198,199]	[171,242]	[108,196,203,216]
Follicular GrzB			[196]	[200]		[196]
Interfollicular GrzB			[196]	[200]		[196]
Granulysin						[216]
Perforin			[196]			[108,196]
Follicular perforin			[196]			[196]
Interfollicular perforin			[196]			[196]
TIA-1		[201]	[196]			[177,196,197,201,203]
Follicular TIA-1			[196,201]			[196,201]
Interfollicular TIA-1			[196]			[196]
Tryptase			[196]			[196]
Follicular tryptase			[196]			[196]
Interfollicular tryptase			[196]			[196]
**CD8^+^ T_regs_**						
CD8^+^FOXP3^+^				[216]		
**CD4^+^ T cells**						
CD4		[201]	[196,205]	[203]	[142,196,202,207]	[52,108,150,177,185,190,197,201,204,205,206,207]
Follicular CD4		[140,201]	[196]	[117]	[196]	[52,190,201,206]
Interfollicular CD4	[140]		[196]	[117]		[52,190,196,206]
**CD4^+^FOXP3^+^ T_regs_**						
FOXP3		[201]	[196,205,209]	[152,206,212,213,214]	[211]	[52,150,168,171,175,183,190,196,197,201,203,205,209,210]
Follicular FoxP3		[205]	[196]	[196,206]	[108,175,205]	[52,117,190,215]
Interfollicular FoxP3		[201]	[140,196]	[117,190,203,215]		[52,196,201,206,211,216,217]
CD8/FOXP3 ratio					[215]	
**Activated Tregs**						
CD4^+^FOXP3^+^PD1^+^TIGIT^+^					[218]	
CD25			[205]		[219]	[203,205,220]
Follicular CD25			[205]		[205]	
**T helper 1 cells**						
T-bet		[140]				
**T helper 17 cells**						
RORγt						[138]
**T cell activation**						
CD27				[220]		[169,172]
CD28				[220]		
CD69		[140]		[213]	[202]	[190]
CD70					[243]	
CD80						[244]
CD86						[244]
CD137						[244]
GITR						[244]
GITRL						[244]
ICOS						[150]
OX40						[244]
OX40L					[244]	
**T cell phenotypes**						
CD4^+^CD8^+^			[193]			[193,216]
CD4^+^CD57^+^						[232]
CD4^+^CD57^+^PD-1^low^					[232]	
CD8^+^CD57^+^					[232]	
CD4^+^PD1^+^						[232]
CD4^+^PD-1^low^					[245]	
CD4^+^PD-1^high^				[246]		[245]
CD8^+^PD-1^low^					[245]	
LAG3^+^TIM3^+^					[247]	
LAG3^+^PD1^+^					[247]	
PD1^+^CXCR5^−^CD27^+^CD28^+^				[220]		
PD1^+^CXCR5^+^CD27^+^CD28^+^				[220]		
PD1^+^CCR4^−^CD27^−^CD28^−^						[220]
PD1^+^CCR4^+^CD27^−^CD28^−^					[220]	
CD8EM/Th1-rich [193]			[193]			[193]
Tfh-rich [193]			[193]			[193]
Exhausted immunophenotypes [240,248]					[240,248]	
**Early-stage differentiation**						
Naïve CD4^+^ T cells				[249]		
Naïve CD8^+^ T cells						[249]
CD45RA				[232]		
CD4^+^CD45RA^+^CCR7^+^				[232]		
CD8^+^CD45RA^+^CCR7^+^				[232]		
CD45RO^−^CCR7^+^				[220]		
**Late-stage differentiation**						
CD4^+^CD45RA^−^CCR7^+^				[232]		
CD45RA^−^CCR7^+^ T memory					[117]	
CD127				[248]		
CD127^+^KLRG1^+^				[248]		

#### 3.5.2. Immune Activation and Exhaustion

Studies have shown that tumor-infiltrating T cells are generally skewed toward an exhausted phenotype in FL, marked by low expression of the co-stimulatory receptors CD27 and CD28 as well as high expression of inhibitory checkpoint molecules such as PD-1, LAG3, and TIGIT. This phenotype ultimately leads to reduced interaction with antigen-presenting cells and impaired T-cell receptor signaling responses. Accordingly, immune activation and exhaustion has been studied thoroughly over time. The expression of T-cell activation markers CD27 and CD28 has been shown with favorable impact [220]. Numbers of PD1-expressing cells have shown contradictory results. Some studies have found PD1 to be associated with a favorable outcome and reduced risk of HT, which may seem intriguing, as PD1 is an immune checkpoint whose ligation inhibits T-cell activation and thereby contributes to immune suppression [117,150,152,196,209,214,235,246,250]. Contrary to this, other studies have associated PD-1 expression with inferior outcomes and an increased risk of transformation [168,195,201,204,209,251,252,253]. Once again, reports also differ depending on intratumoral compartmental localization. Moreover, inferior outcomes have also been reported based on the expression of other inhibitory markers such as TIM3, LAG3, and TIGIT, while CTLA4 and IDO1 have been reported without prognostic value only [199,244,247,253,254,255].

**Table 11:** Immune exhaustion.

	Reported Risk of Transformation			Reported Prognostic Value		
	Favorable	Inferior	None	Favorable	Inferior	None
PD-1	[214]	[251]	[196,209]	[196,214,250]	[168,195]	[52,108,150,209,212,220,244,247,251,252]
Follicular PD-1	[209]	[201]	[196,251]	[117,150,152,196,209,246]	[251,252]	[52,171]
Interfollicular PD-1		[209]	[196]	[235]	[204,209]	[52,117,171,196,216,217,246]
PD-L1						[204,244,256]
Follicular PD-L1		[201]				[201]
Interfollicular PD-L1						[216]
PD-L2						[244]
TIM3					[199]	[244]
Follicular TIM3						[117]
Interfollicular TIM3						[117]
LAG3		[253]			[247,253]	[244,256]
LAG3^+^PD-1^+^		[253]				
TIGIT					[254]	
CTLA4						[244]
IDO1			[255]			[255]
Trp						[257]
Kyn					[257]	
Galectin-9						[244]

**Table 12:** Natural killer cells.

	Reported Risk of Transformation			Reported Prognostic Value		
	Favorable	Inferior	None	Favorable	Inferior	None
CD56			[196]			[196]
Follicular CD56			[196]			[196]
Interfollicular CD56			[196]			[196]
CD56/MS4A4A ratio					[246]	
CD57		[201]	[140,196]		[202,232]	[177,196,197,201]
Follicular CD57		[201]	[196]		[108]	[196,201]
Interfollicular CD57			[196]			[196]

#### 3.5.3. Follicular Dendritic Cells

Non-neoplastic stromal cells, including follicular dendritic cells (fDCs), play an important role in the pathogenesis of FL. Especially, the fDC markers CD21 and CD23 have received much attention in FL research, yielding contradictory results, with reports on favorable, inferior, as well as no impact on transformation and outcomes regarding both markers [61,108,140,142,188,190,201,209,212,239,258,259,260].

In addition to fDCs, the most prominent stromal cellular subpopulation in FL is follicular reticular cells (F_RC_s). These cells are present in the T cell region around the follicle and secrete different components of extracellular matrix [261]. They contribute by secreting IL-4, CXCL12, CXCL13, IL-7, and BAFF. While BAFF itself was reported without impact on outcome, its B cell-expressed counterpart, BAFF receptor (BAFFR), was reported with an inferior impact on outcome [262,263].

**Table 13:** Dendritic cells.

	Reported Risk of Transformation			Reported Prognostic Value		
	Favorable	Inferior	None	Favorable	Inferior	None
CD21	[258]	[140,201]	[209]		[201]	[142,190,209,258]
Follicular CD21					[201]	
CD23			[140]	[259]	[108,190]	[61,188,212,239,260]
CD11c			[209]			[209,211,264]
Follicular CD11c					[211]	
CD1a						[211]
CD83						[211]
Ki-M4p						[260]
PU.1				[169]		[172]
**Plasmacytoid dendritic cells**						
CD123				[233]		[197,211]

#### 3.5.4. Tumor-Associated Macrophages

Macrophages have long been investigated in relation to FL, with in vitro studies reporting a dependency of FL cells on macrophages for survival and proliferation [265]. In this regard, a high lymphoma tissue content of CD68^+^ tumor-associated macrophages (TAMs) has been associated with a poor outcome among FL patients in several studies [142,168,177,266,267,268,269]; however, controversially, also with several reports of favorable outcomes [75,158,201]. It should also be noted that quite a few studies did not find any association with either transformation or outcome [52,108,140,171,177,190,196,197,203,204,209,235,264,268,269,270]. Related to outcome, especially, interfollicular localization of CD68^+^ macrophages has been reported with inferior outcomes [175,196,200]. Oppositely, when investigating TAMs in association with the risk of transformation, only one study found the follicular localization of CD68 to predict transformation, while the remaining studies found no association [140,177,196,201,209,269]. The reported inferior outcome was particularly shown by the presence of a protumoral phenotype (so-called M2-like macrophages), which was defined in analyses based on the markers CD163, CD206, and CSF-1R, which are all prominent in protumoral-type macrophages [240,246,264,268,271]. On the other hand, also with the protumoral phenotype, one study reported a favorable outcome, while several studies found no difference in outcomes [52,108,204,216]. Only CSF-1R has been reported with an association with subsequent transformation [271].

Differences in these reports may be considered based on the type of administered treatment. The CD20-targeting antibody rituximab can be used by TAMs to facilitate antibody-dependent cellular cytotoxicity and phagocytosis [265,272]. Clinical studies performed in the rituximab era have yielded conflicting results on the effect of macrophages, with variable outcomes based on the type of regimen used. Thus, the impact of TAMs on FL prognosis may depend on the administered treatment. Moreover, novel agents targeting the CD47/SIRPα axis are under development, which makes the SIRPα protein an interesting biomarker for investigation. Malignant cells, including FL cells, upregulate the surface marker CD47, which interacts with the macrophage receptor SIRPα to avoid phagocytosis [86]. While investigated in only a limited number of studies, associations between a follicular localization of SIRPα and outcome have been reported [117]. Additionally, another paper showed that CD14^+^SIRPα^+^ co-expression in lymphomas also resulted in inferior outcomes [86].

As already exemplified, the prognostic value of different macrophage markers also seems dependent on the choice of treatment. Contemporary studies have shown conflicting results when rituximab was incorporated into treatment regimens. Among others, this was reported by Kridel et al. in a study that demonstrated high numbers of CD163^+^ macrophages to be independent predictors of improved progression-free survival in patients allocated to anthracycline-based regimens [268].

**Table 14:** Macrophages.

	Reported Risk of Transformation			Reported Prognostic Value		
	Favorable	Inferior	None	Favorable	Inferior	None
CD68			[140,177,196,209,269]	[91,212,236]	[142,168,177,266,267,268,269]	[52,108,171,190,196,197,203,204,209,235,264,268,270]
Follicular CD68		[201]	[196]	[91,246]	[266]	[52,117,175,183,188,190,196,201,215]
Interfollicular CD68			[196]		[175,196,200]	[52,91,117,183,188,190,215,216,246,266]
CD14			[209]		[86,202]	[209]
Follicular CD14		[209]				[117,209]
Interfollicular CD14	[209]					[117,209]
SIRPα						[86]
Follicular SIRPα					[117]	
Interfollicular SIRPα						[117]
CD14^+^SIRPa^+^					[86]	
**Pro-inflammatory/** **M1-like macrophages**						
iNOS						[108]
**Anti-inflammatory/** **M2-like macrophages**						
CD163				[52]	[264,268]	[108,204]
Follicular CD163						[52]
Interfollicular CD163						[52]
CD163/CD8 ratio					[246]	
CD206					[240]	
Interfollicular CD206						[216]
CSF-1R		[271]			[271]	
Follicular CSF-1R		[271]			[271]	

#### 3.5.5. Angiogenesis

Among classical hallmarks of cancer, angiogenesis plays an important role in providing nutrition and oxygen in both solid tumors as well as hematopoietic tumors, and thus, the significance of vessel density is not to be neglected. In FL, microvessel density or counts have been investigated often as a proxy for angiogenesis assessment counted by the expression of endothelial markers, either CD31 or CD34. In general, increased lymph node vascularization, as indicated by these two markers, has been reported to confer inferior impact on transformation and outcome, with only one contradictory study reporting a favorable outcome [151,264,269,273,274,275]. In the study by Farinha et al., tumor-to-vessel density was further associated with the numbers of lymphoma-associated macrophages, which the group had previously also found to be associated with inferior outcomes in FL, as also described above [177,269]. Several other angiogenic markers have also been investigated, yielding favorable outcomes associated with the expression of estrogen receptor α, inferior outcomes correlated with VEGF expression, as well as inferior risk of transformation with expression of the VEGF receptor KDR (also known as VEGFR-2) [276,277].

**Table 15:** Tumoral vascularization.

	Reported Risk of Transformation			Reported Prognostic Value		
	Favorable	Inferior	None	Favorable	Inferior	None
**Increased microvessel density**						
CD31					[151,264]	[147]
CD34		[269]		[273]	[269]	[264,274]
Follicular CD34		[275]				[275]
Interfollicular CD34		[275]			[275]	
**Angiogenesis**						
Estrogen receptor α				[276]		
FLT-1						[277]
FLT-4						[277]
KDR		[277]				[277]
LYVE-1						[264]
Podoplanin						[264]
PROX1						[264]
VEGF					[277]	
VEGF-C						[277]
VWF						[264]

#### 3.5.6. Energy Metabolism and Vitamin D Insufficiency

It is well established that cancer cells often undergo metabolic reprogramming to adapt to their increased energy requirements [278,279,280]. Related to this, studies have reported on biomarkers in FL related to glucose metabolism, including protein levels of aldolase A, glyceraldehyde-3-phosphate dehydrogenase (GAPDH), and glucose transporter 1 (GLUT1) [240,281]. Whether the process of transformation is indeed dependent on this pathway requires further investigation; however, the reported increased expression levels of GLUT1, affecting glucose uptake, as well as aldolase A and GAPDH, affecting glucose processing, could suggest a general increase in the glycolytic metabolism in FL patients at risk of subsequent transformation. Regardless of the preferred metabolic state of tFL cells, the notion of a generally increased glycolysis in transformation-prone FL tumors is in itself rather interesting.

Vitamin D has been proposed to exert anti-cancer and anti-metastatic effects, with actions primarily mediated through its metabolized hormonal form, 1,25-dihydroxyvitamin D (1,25(OH)2D), classically regulating pathways involved in calcium and phosphorous homeostasis; however, 1,25(OH)2D has also been identified as a promising anti-cancer agent affecting tumorigenic effects such as proliferation and apoptosis [282,283]. In accordance with this, two studies have reported on vitamin D insufficiency in FL, both linking low vitamin D levels with inferior outcomes [284,285]. Interestingly, recent evidence has also identified 1,25(OH)2D as a regulator of energy metabolism in cancer cells through the inhibition or reversal of altered glucose metabolism, including reducing glucose uptake into cancer cells via glucose transporters, of which GLUT1 was recently associated with inferior outcomes in FL by Deng et al. [240].

Furthermore, the addition of rituximab as a standard therapy has significantly improved the outcome of FL patients, regarding both survival and risk of transformation [101,286,287]. One mechanism of action of rituximab is antibody-dependent cellular cytotoxicity (ADCC), which may be mediated by different types of effector cells, including neutrophils, macrophages, and natural killer (NK) cells [99,100,101,102]. However, interestingly, an association of ADCC and vitamin D3 has been described for both macrophages and NK cells, with vitamin D3 deficiency impairing the rituximab–ADCC function [288]. Possible mechanisms or signaling pathways of vitamin D3 to affect NK cell-mediated ADCC have not yet been investigated, and thus, further investigation of the vitamin in FL is definitely called for.

#### 3.5.7. Cell Death

Most cases of FL overexpress the anti-apoptotic BCL2 protein as a result of the t(14;18). Furthermore, several studies have investigated inappropriate regulation of the apoptotic pathway in FL. Related to transformation, apoptotic proteins including BAX, BCL-rambo, BCL-xL, CASP3, and MCL1 have shown an inferior association [289]. This has been accompanied by inferior outcomes with increasing levels of BCL-xL, MCL1, BCL2/BAK, and BCL2/BAX ratios [172,176,184], while the level of YY1 was reported with a favorable outcome [290]. Interestingly, these reports suggest apoptotic deregulation beyond the t(14;18), which makes this pathway an interesting focus for future research. Although traditionally regarded as an important barrier to tumorigenesis, apoptosis may also instigate proliferation-inducing paracrine effects, resulting in accelerated cell growth [289,291,292]. Additionally, oncogenic effects associated with apoptosis also include the phagocytosis of apoptotic cells by macrophages, which has been proposed to contribute to an immunosuppressive TME though the removal of inflammatory signals [289,292]. Indeed, exact mechanistic studies elucidating expression levels in specific intratumoral cell types are warranted as the named proteins include both anti- and proapoptotic properties.

**Table 16:** Other microenvironmental factors.

	Reported Risk of Transformation			Reported Prognostic Value		
	Favorable	Inferior	None	Favorable	Inferior	None
**Other leukocyte markers**						
AID		[72]				[262]
BAFF						[263]
BAFFR					[263]	
BLIMP1						[171]
BTLA			[152]	[152]		
Follicular BTLA				[152]		
Interfollicular BTLA				[152]		
CD30					[172]	
CD32B (FcγRIIB)						
Follicular CD32B						[117]
Interfollicular CD32B						[117]
CD38						[172]
CD44			[293]			
Follicular CD44		[293]				[293]
CD44s					[274]	
CD70						
Follicular CD70						[117]
Interfollicular CD70						[117]
CD9					[294]	
ETV1						
Follicular ETV1	[295]				[295]	
Interfollicular ETV1	[295]			[295]		
HLA-DR				[242]		[190,211]
Follicular HLA-DR						[211]
LMO2						[54]
NAMPT						
Follicular NAMPT	[295]			[295]		
Interfollicular NAMPT			[295]	[295]		
PI3Kδ					[294]	
PMCH						
Follicular PMCH		[295]		[295]		
Interfollicular PMCH	[295]			[295]		
**T cell immunological synapse**						
Filamin A						
Follicular Filamin A						[234]
Interfollicular Filamin A						[234]
Itk						
Follicular Itk						[234]
Interfollicular Itk						[234]
RAB27A				[234]		
Follicular RAB27A				[234]		
Interfollicular RAB27A						[234]
**Complement inhibitors**						
CD46						[186]
CD55						[186]
CD59						[186]
**NF** **κB activity**						
p65 (RelA)					[91]	[296]
pRB						[172]
**Cytokines/chemokines**						
CCR1						[297]
CXCL13			[209]			[209]
IL-10						[108]
IL-12A						[108]
IL-17A						[108]
IL-17F					[108]	
IL-2						[108]
IL-21R					[298]	
TGFB1						[108]
TGFBR1						[108]
**The cytoskeleton and cellular migration**						
CDK6						[172]
FilGAP					[299]	
Integrin B2						[299]
RHAMM		[293]			[293]	
Follicular RHAMM		[293]			[293]	
CD44/RHAMM ratio, low		[293]			[293]	
Vimentin	[300]	[181]			[181]	
**G protein-coupled signals**						
GNA13					[301]	
Rac1						[299]
**Metalloproteinases**						
MMP2			[302]			[302]
MMP9			[302]			[302]
TIMP1			[302]			[302]
TIMP2			[302]			[302]
**Signal transduction**						
EPHA1				[147]		
pJAK2				[303]		
SOCS3		[304]			[304]	
STAT5a				[134]		
pSTAT5				[303]		
STAT1						[270]
CD68^−^STAT1^+^					[270]	
**Peroxiredoxins**						
Peroxiredoxin, total				[305]		
PRDX1						[305]
PRDX2						[305]
PRDX3						[305]
PRDX4						[305]
PRDX5						[305]
PRDX6						[305]
**Oxidative stress**						
OHdG						[306]
Gamma-GCS						[306]
Thioredoxine						[305]
Nitrotyrosine						[305]
Superoxide dismutase						[306]
**Cell cycle**						
ACPI						[307]
BMI1					[308]	
ECT2						[299]
CDK2						[172]
Cyclin A						[143,172]
Cyclin B1				[143]		[172]
Cyclin D3						[172]
Cyclin E					[172]	
E2F6					[172]	
EZH2						[77]
MDM2					[61,172]	
MYC			[85]		[162]	
p18						[172]
p21					[172]	
p27						[91,172]
p53		[302]	[258]		[302,309]	[52,172,174]
Follicular p53						[52]
Interfollicular p53						[52]
P-glycoprotein			[258]			[309]
S100						[211]
SKP2						[172]
**Glucose metabolism**						
GLUT1					[240]	
Aldolase A	[300]	[281]			[281]	
GAPDH	[300]	[281]			[281]	
ATP synthase δ	[300]					
**Cell death**						
14-3-3γ						[184]
Akt						[184]
pAkt						[184]
Aurora A						[184]
BAD						[184]
BAK						[184]
BAX		[289]				[172,173,184]
BCL-rambo		[289]				[184]
BCL-x						[173]
BCL-xL		[289]			[172]	[177,184]
CASP3		[289]				[184]
CASP3a						[172]
cCASP3						[184]
MCL1		[289]			[176]	[184]
PARP						[184]
cPARP						[184]
SMAC						[184]
Survivin						[172,184]
XIAP						[184]
BCL2/BAK ratio					[184]	
BCL2/BAX ratio						[184]
YY1				[290]		
YY1/PLK1 interaction					[310]	
**Metabolomics**						
Metabolomic profile [311]					[311]	

## 3.6. Soluble Protein Measurements

Soluble proteins measured in body fluids (i.e., serum, plasma, urine, etc.) have mainly focused on differences in outcome rather than risk of transformation, with the majority of studies focusing on immune-related components. One soluble protein often analyzed is the soluble form of IL-2R and IL-2Rα, generally yielding an inferior impact on transformation and outcome [219,312,313,314,315,316,317,318,319,320,321]. Other interleukins investigated that are related to inferior outcomes include IL-1RA, IL-4, IL-6, and IL-12 [315,318,322,323]. Likewise, other immune-related proteins correlated with inferior outcomes include TNFα, CFHR3, CXCL9, CCL22, and APRIL, while CFHR1 and CCL19 were associated with a favorable outcome [148,315,318,324,325,326].

**Table 17:** Soluble protein markers.

	Reported Risk of Transformation			Reported Prognostic Value		
	Favorable	Inferior	None	Favorable	Inferior	None
APRIL					[324]	
BAFF						[324]
CA-125					[327]	
CCL17						[325]
CCL19				[148]		
CCL22					[325]	
CCL3						[318]
CCL4						[318]
CCL5						[318]
CFH						[326]
CFHR1				[326]		
CFHR3					[326]	
CXCL9					[318]	
CXCL10						[318]
EGF						[318]
Eotaxin						[318]
HGF					[318]	
IFN-α						[318]
Ig free light chains					[328]	
IL-1RA					[318]	
IL-2						[318]
IL-2R		[319]	[315]		[219,312,313,314,315,316,317,318]	[329,330]
IL-2Ra					[320,321]	
IL-4					[322]	[318]
IL-6			[315]		[315]	
IL-8						[318]
IL-10						[318]
IL-12					[318,323]	
IL-13						[318]
LR11					[331]	
MCP1						[318]
Selenium				[332]		
Thymidine kinase 1 (TK1)					[333]	
TNF-α			[315]		[315]	
Triiodothyronine (T3)				[334]		
Vitamin D insufficiency						
Low vitamin D					[284,285]	
Cholesterols						
High-density lipoprotein cholesterol					[335]	
Low-density lipoprotein cholesterol						[335]

### Circulating Tumor DNA

An area of investigation that has become increasingly relevant in recent years is the detection of circulating tumor DNA (ctDNA) measured from samples of blood or urine, allowing for a less invasive monitoring of disease. While not many studies have yet focused on cell-free DNA (cfDNA) or ctDNA in FL, studies were identified reporting inferior outcomes with higher proportions of ctDNA [30,31,336,337]. The mutational status of a few specific genes has been studied, of which inferior outcomes were associated with *BCL2*, *EP300*, *KMT2D*, *STAT6,* and *TP53* mutations [71,338]. As FL patients are often diagnosed with disseminated disease, ctDNA measurements may be especially relevant, as this may constitute a pool of genetic information originating from multiple clones and tumoral sites, thus often considered an integrator of the mutational heterogeneity.

**Table 18:** Cell-free DNA.

	Reported Risk of Transformation			Reported Prognostic Value		
	Favorable	Inferior	None	Favorable	Inferior	None
**ctDNA**						
High proportion of ctDNA					[30,31,336,337]	
Detectable ctDNA mutations					[338]	
Specific genetic mutations in						
*BCL2*					[71]	
*CARD11*						[338]
*CREBBP*						[30,338]
*EP300*					[338]	
*KMT2D*					[338]	
*PCLO*						[338]
*STAT6*					[338]	
*TP53*					[30]	
**cfDNA**						
High proportion of cfDNA					[31,71]	[339]
cfDNA, cell-free DNA; ctDNA, circulating tumor DNA.						

## 4. Discussion

We systematically assessed the current literature for research on the impact of molecular biomarkers in FL transformation and outcomes, providing a comprehensive overview of studies conducted within the past four decades. In this time period, biomarkers have been analyzed in the hundreds, underscoring the persistent challenge of risk anticipation of FL patients. An accurate assessment of transformation risk already at FL diagnosis might allow low-risk patients to avoid unnecessary interventions and frequent hospital visits, decrease worry and anxiety, and thus, have a chance of a better general quality of life. At the same time, high-risk patients might undergo additional monitoring and treatment to improve the poorer outcomes observed in patients with transformed FL.

The majority of the included studies were published within the past two decades. The rapid pace of methodological advancements as well as scientific discoveries in FL will undoubtedly constitute a challenge in prioritizing the most clinically relevant and essential prognostic/predictive biomarkers [340]. This is further underscored as the information currently available has often conferred redundant or conflicting results. This overview gives rise to further validation studies of several putative markers, ultimately providing insight into whether or not these hold promise as clinically applicable biomarkers.

Notable differences in the reports on predictive and/or prognostic value were observed for several biomarkers. Over time, criteria for diagnosis and classification have changed, which may be reflected in the reported findings. Differences in the definition of transformation or time-related endpoints also vary between studies, e.g., with some studies including only biopsy-proven histological transformation, while others include also clinically suspected transformation. Furthermore, researchers have faced several challenges in studying transformation biology. Methodological variability in the sample sources (archival tissue, fresh-frozen tissue, etc.) and types of controls could be a source of confounders, limiting the direct comparison of results. Furthermore, the available pool of archival tissue is limited, particularly when seeking paired low-grade as well as transformed biopsies, with time being an important aspect in a disease with survival measured in decades. Many clinical studies of biological markers tend to use already clinically applicable methodologies such as immunohistochemical staining. Although already routinely used in clinical pathology, it is often rather difficult comparing results from different studies due to high inter-observer variations. Moreover, before clinical implementation, it is also important to reach agreement on the cutoff values for dichotomizing biomarker expression. Thus, discrepancies between reports might also be attributed to technical differences between studies.

Interestingly, several studies found prognostic molecular differences depending on gender, i.e., a biomarker predictive of transformation or outcome in either men or women, but not in both [49,109,252]. Generally, more research has focused on gender differences in lymphoma, and a recent study reported better outcomes among female FL patients than male patients [341,342]. Thus, additional research into biological gender differences in FL is warranted.

The present review was based on a rather broad search strategy, with the purpose of avoiding overlooking potentially important papers. Nevertheless, in the attempt to narrow the search, commonly described markers were excluded (e.g., β2-microglobulin, hemoglobin, LDH, Ki67, t(14;18)/*BCL2* rearrangements). With this broad search, a large number of articles were manually screened and reviewed, which is why it is likely that some papers might have been missed. Furthermore, systemic reviews usually include a manual in-depth quality assessment of all included articles. However, due to the large number of articles included in the present study, this assessment was waived, which might introduce the risk of including lower-quality papers. However, all included papers were from peer-reviewed journals. Furthermore, upon data extraction, if the authors questioned the quality of a paper, the paper was discussed among the reviewers, thereby reducing the risk of including lower-quality papers.

This review has provided an overview of the current literature investigating molecular biomarkers in transformation and outcomes in FL. Notably, these must be considered two different outcomes; however, currently, transformation remains the leading cause of FL-related mortality. The present review was constructed with the aim to guide new research ideas, and thus, we included both the transformation and prognostic endpoints to provide an overview of potentially relevant markers for research in the future. A multitude of factors have been investigated in the attempt to understand and predict outcomes in FL and to anticipate patient disease course at the time of diagnosis. However, these largely remain inaccessible in daily practice. Further adequate studies are certainly warranted, possibly investigating a multimarker approach in combination with clinical data.

## Data Availability

Data may be shared upon reasonable request to the corresponding authors.
